# Surgical Access to a Complex Composite Odontoma via Sagittal Split Osteotomy of the Mandible

**DOI:** 10.7759/cureus.1915

**Published:** 2017-12-06

**Authors:** Pradeep J Christopher, Senthilnathan Periasamy, Poorna Devadoss, Santhosh P Kumar

**Affiliations:** 1 Oral and Maxillofacial Surgery, Thai moogambigai dental college & hospital; 2 Oral and Maxillofacial Surgery, Saveetha Dental College, Saveetha University, Chennai; 3 Orthodontics and Dentofacial Orthopaedics, Thai moogambigai dental college & hospital

**Keywords:** sagittal split osteotomy, mandible, surgical access, complex composite odontoma

## Abstract

This case report presents the removal of complex composite odontoma in a young patient in the right body of mandible via the unilateral sagittal splitting of the mandible. This article shows that sagittal split osteotomy of the mandible can be very useful to access various pathologies in the body, angle, and ramus of the mandible and to navigate lesions that are in proximity to the inferior alveolar nerve. This technique also helps in avoiding postoperative morbidity when compared to other conventional surgical approaches. It can be used to remove large cysts, benign non-infiltrative tumours of the mandible, odontogenic myxoma, large odontoma, and deeply impacted lower third molars.

## Introduction

The odontomas are the most common benign odontogenic tumours. Radiologically and histologically, there are two types of odontomas: complex and compound. The complex odontoma consists of a mass of irregularly arranged tissues and does not resemble any tooth structure. The compound odontoma has a collection of morphologically recognizable teeth in the tissue mass [1]. They are usually asymptomatic but are sometimes associated with pain in the region where they are present. They are mostly diagnosed incidentally during routine radiographic examinations. They remain constant in size with increasing age. These are considered to have very limited potential for growth. However, in some cases, they have attained larger dimensions during active growth periods.

Small and superficially placed odontomas can be easily accessed by a simple intraoral approach. Large and deeply placed odontomas in the ramus or body of mandible require either an extraoral approach or necessitate removal of large amounts of bone, thus resulting in mandibular fracture or damage to the inferior alveolar nerve. Sagittal split osteotomy (SSO), introduced by Rittersma and Van Gool in 1979, is an excellent technique for accessing various pathologies located deep in body or ramus of the mandible and in close proximity with the inferior alveolar nerve [2]. This technique can also avoid various complications associated with the conventional approaches.

In this report, we present a case of symptomatic odontoma in the molar area of the right mandible which was accessed via SSO of the mandible and was treated by enucleation.

## Case presentation

**Figure 1:**
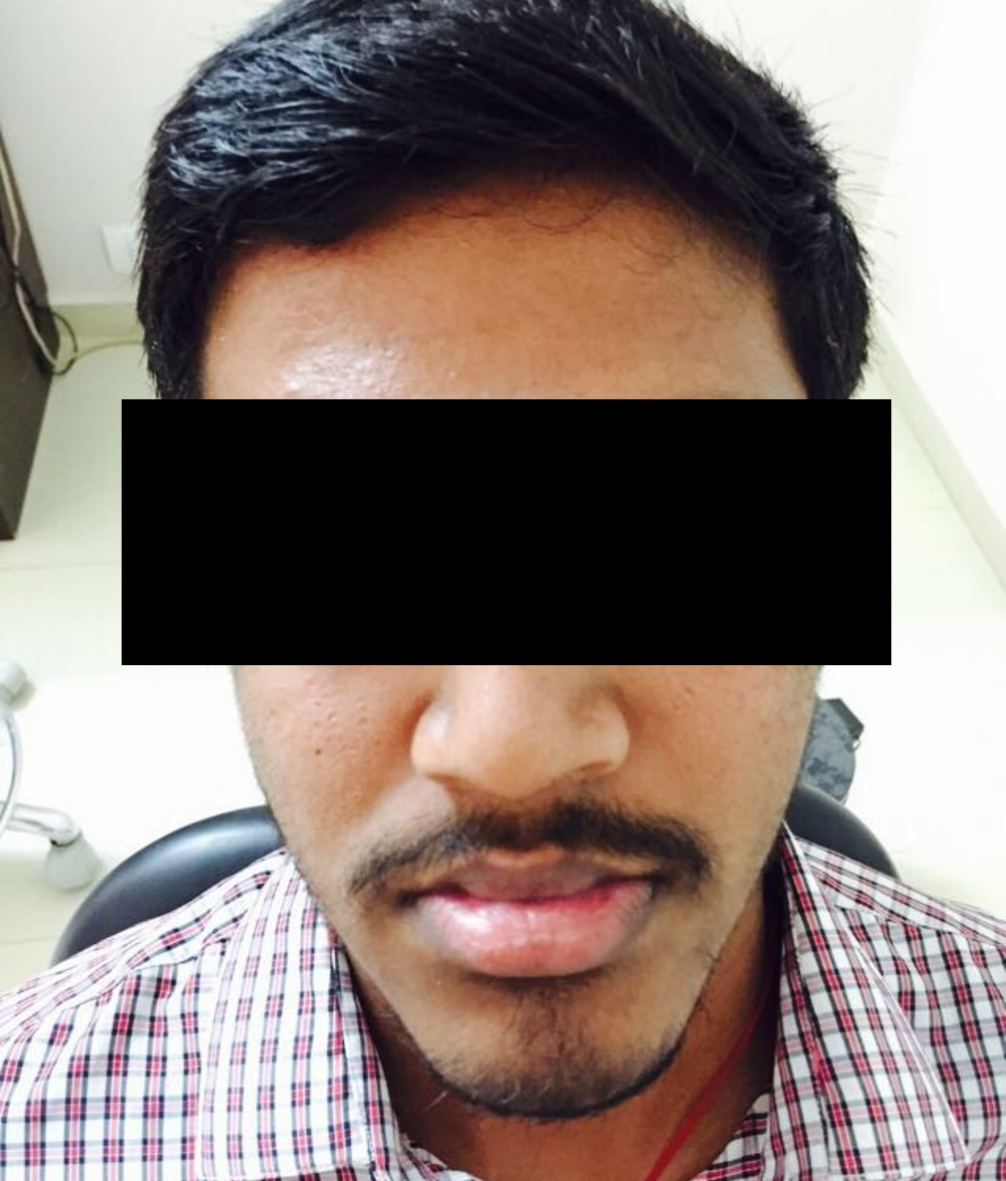
Extraoral view

**Figure 2:**
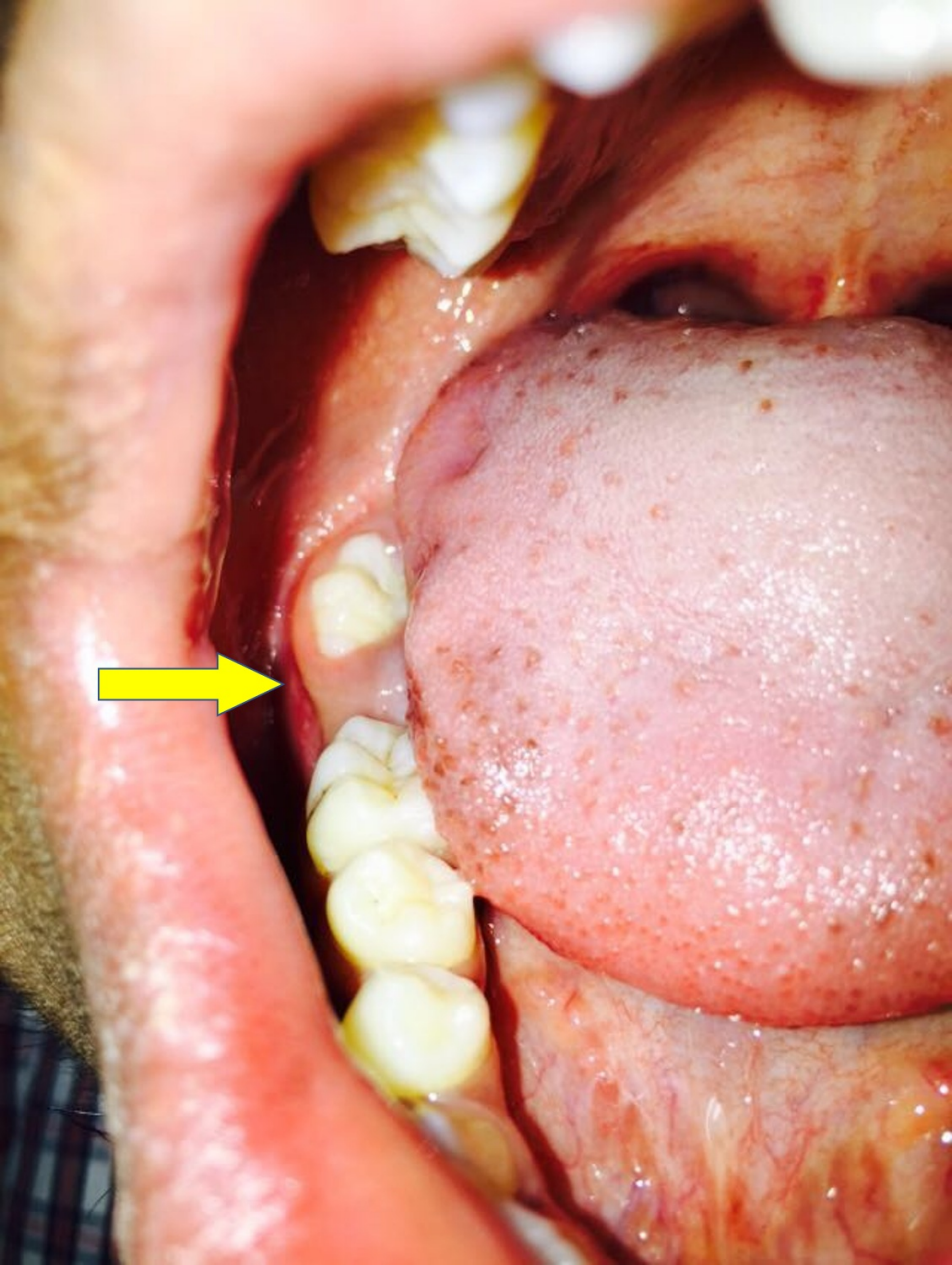
Intraoral view of odontoma

**Figure 3:**
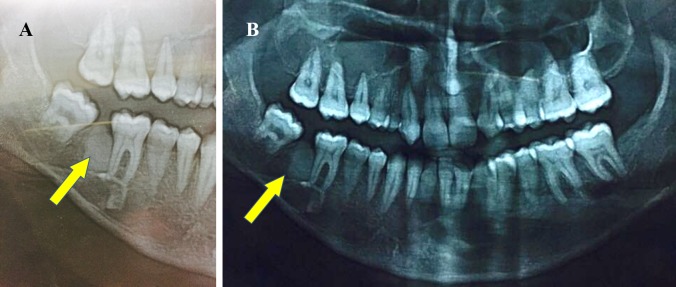
Orthopantomograph showing odontoma in the 47 region

A 28-year-old male patient reported to the Department of Oral and Maxillofacial Surgery, Saveetha Dental College and Hospitals, Chennai, India, with a chief concern of pain over the lower back tooth region on the right side for the past month. The patient’s past medical history was within normal limits. Extraorally, no swelling was seen on the right side of the face, and sensation in the distribution of the right mental nerve was normal (Figure 1). Intraoral examination revealed missing 47 and partially erupted 48, and there was no evidence of buccal or lingual cortical expansion of the mandible in the posterior region (Figure 2). A panoramic radiograph revealed a radioopaque mass between 46 and 48, and the lesion was surrounded by a thin radiolucent line (Figure 3). Based on the clinical signs, symptoms, and the radiographic evidence, the lesion was diagnosed as complex composite odontoma.

**Figure 4:**
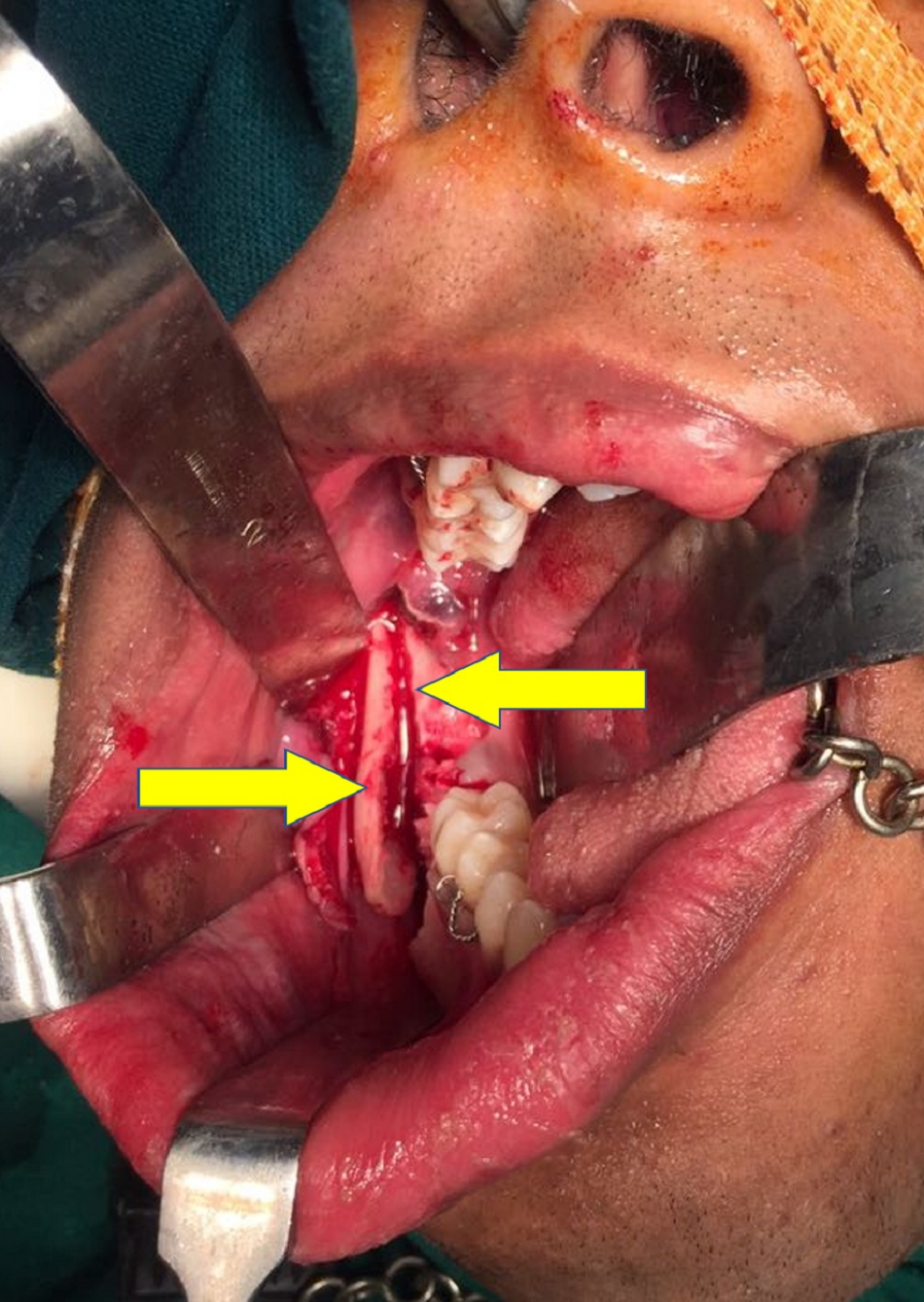
Sagittal split of mandible

**Figure 5:**
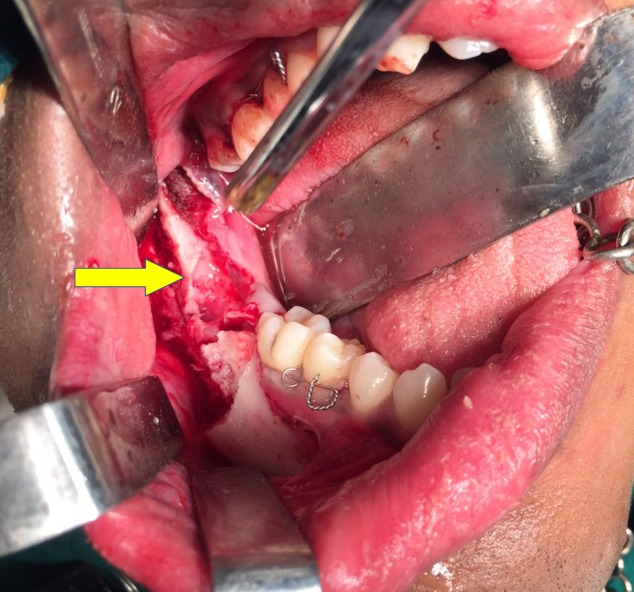
Odontoma exposed via sagittal split osteotomy

**Figure 6:**
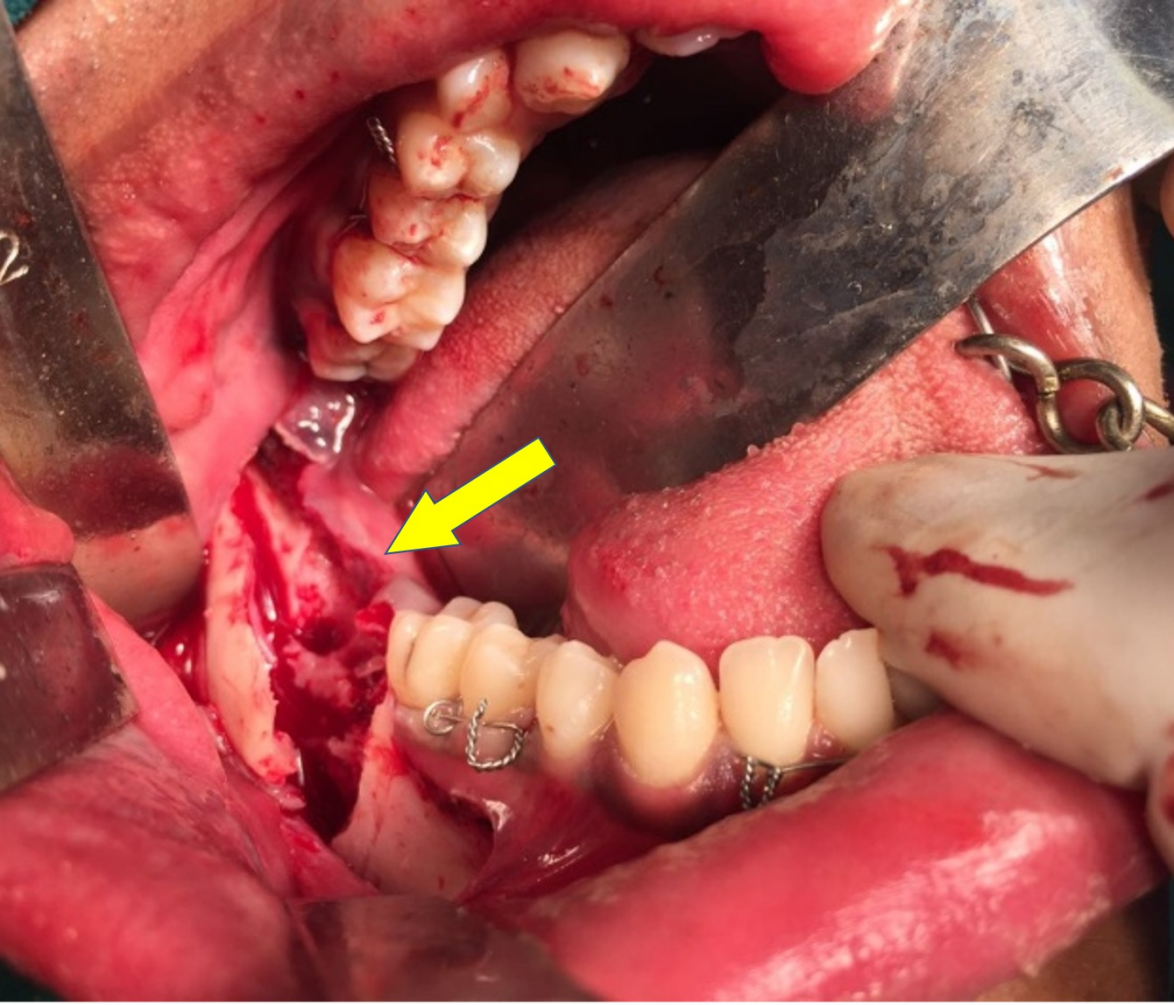
Surgical site after odontoma removal

**Figure 7:**
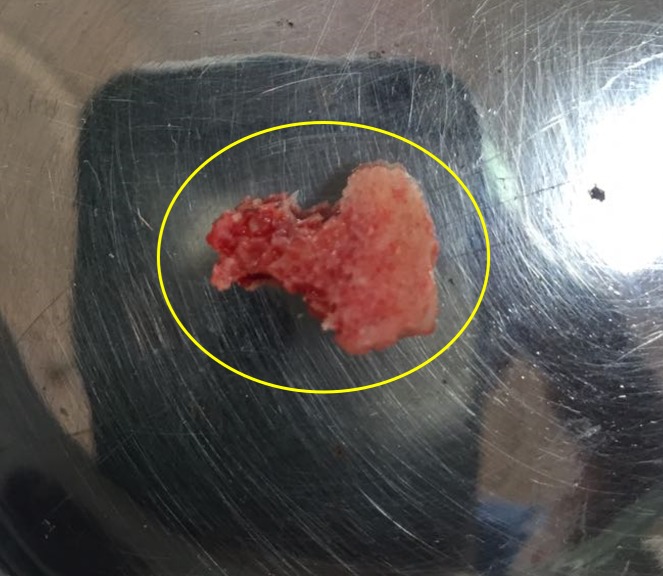
Excised specimen

**Figure 8:**
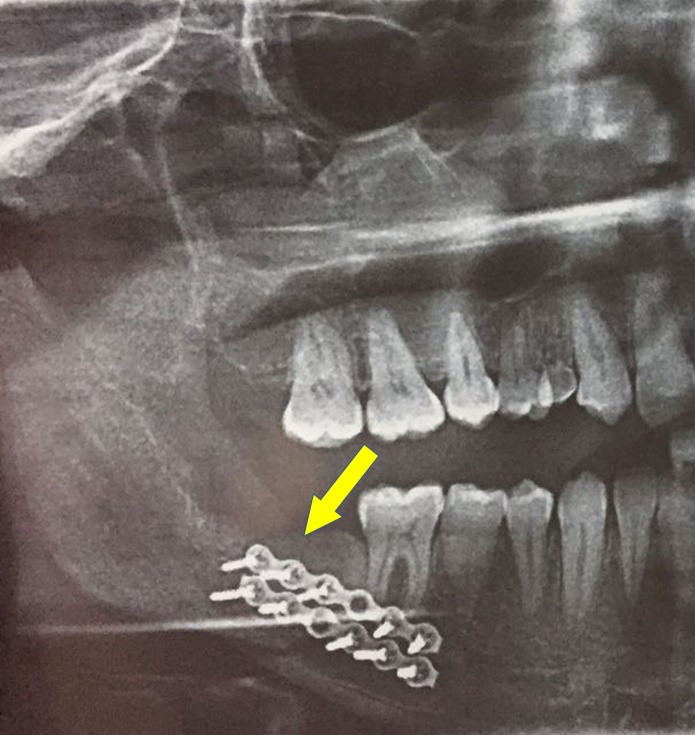
Six months postoperative orthopantomogram showing excellent wound healing

The patient was scheduled for removal of the odontoma using the sagittal split osteotomy approach as this technique would provide adequate surgical access to the lesion as well as preserve the buccal and lingual cortices. Standard SSO of the mandible was done unilaterally on the right side under general anaesthesia (Figure 4), and the odontoma was exposed between the buccal and the lingual cortices (Figure 5). The odontoma was removed by enucleation, preserving the inferior alveolar nerve without intraoperative complications (Figure 6) and the specimen was sent for histopathological examination (Figure 7). Extraction of 48 was done. Both the cortices of the mandible were kept in normal anatomical approximation, and osteosynthesis was achieved by using two miniplates and screws. The wound was closed, and recovery was uneventful. Postoperatively, there was no paresthesia in the chin and lip area on the right side. The patient was put on intermaxillary fixation for two weeks using eyelet wiring. Histopathology report of the specimen revealed a haphazard arrangement of enamel, dentin, cementum, and dental pulp consistent with a diagnosis of complex composite odontoma. Clinical and radiographic examination six months after surgery showed good results without any complications (Figure 8).

## Discussion

The accepted treatment for symptomatic odontoma is enucleation [1]. In certain cases, there are various factors that affect the treatment plan and limit complete enucleation. These factors can broadly be divided into anatomic factors, access factors, and size of the lesion. The anatomic factors include the proximity of the adjacent tooth structures and nerve components such as inferior alveolar nerve (IAN). One important factor that determines the method of surgical access to the structure is the location of the lesion as it may be too deep or lingually placed. There are chances of fracture in cases where the size of the odontoma is large and located too close to inferior border thus requiring greater removal of bone for complete enucleation of the lesion.

There are various surgical techniques for removing large benign lesions of the mandible [3]. They are:

1. The intraoral buccal approach in which the buccal cortical plate is removed and the lesion is adequately exposed before enucleation.

2. The intraoral lingual approach in which the lingual cortical plate is fractured and the lesion is exposed. Care is taken to dissect the flap along with the periosteum, keep the instrument close to the bone, and prevent any perforation due to the proximity of the lingual nerve.

3. Segmental osteotomy in which extraoral submandibular incision is given and partial resection of the bone is done.

4. Unilateral sagittal split osteotomy of the ramus or mandibular body along with its modifications is another surgical approach to the lesions of the mandible.

Intraoral buccal or lingual approach requires removal of a large amount of cortex (corticotomy) for exposure of the pathology and to obtain adequate access thus weakening the mandible. Corticotomy may hence sometimes need to be supplemented with grafting procedures to prevent fracture of the mandible postoperatively. It is not suitable for removal of large lesions in the mandible. The lingual approach might lead to lingual paresthesia if the plane is not kept subperiosteally. Segmental resection through extraoral approach leaves a bony defect and requires bone grafting.

The sagittal splitting of the ramus or body of the mandible through intraoral approach introduced by Trauner and Obwegeser is a well-established method for correcting dentofacial deformities [4]. Since then, many modifications of this procedure have been performed [5-7]. The sagittal split osteotomy was first introduced for the removal of a large tumour of the mandible in 1979 by Rittersma and Van Gool [2]. Barnard in 1983 [8] presented a case in which sagittal splitting of the mandible was used to gain access for removal of a large complex composite odontoma [8]. Since then it is considered as one of the methods for the removal of deeply seated lesions of the mandible. Several others have used this procedure to access various pathologies in the mandible body/ramus region [9].

SSO has also been used for correction of dentoalveolar deformities, malocclusion and in pre-prosthetic surgery.

There are various noted advantages to using the sagittal split osteotomy for surgical access in the removal of lesions of the mandible. The sagittal split osteotomy is carried out intraorally, thus avoiding an external scar, and is more aesthetic when compared to the extraoral approach. This also prevents injury to the terminal branches of the facial nerve which could be damaged during the exposure of the mandible for osteotomy using extraoral approach. The SSO can be used to remove lesions near the IAN as there is adequate and direct visibility of the canal, hence preventing permanent damage to the neural structure. The visibility of the inferior alveolar nerve and canal is diminished in the extraoral approach making it a relatively bad option with deeply placed or inferiorly placed lesions of the mandible. One factor to be considered is that even with SSO, there is minimal IAN dysfunction. Paresthesia is present in about 34% of cases after four days after surgery, but it drastically reduces to about only 8% after six months. The fact that the paresthesia in the lower lip due to SSO is not very bothersome to the patients in comparison to the paresthesia of the lingual tissues that occurs in the intraoral lingual approach. Various modifications of the SSO described recently can also be used for removal of lesions in mandible that present with thinning of the cortices of the mandible.

SSO is technically more difficult than corticotomy but avoids discontinuity of mandible, thereby preventing the fracture of mandible intraoperatively and postoperatively. It is more preferred in the removal of deep-seated and large pathologies of the posterior mandible. One drawback of the procedure is that it requires plating and may require maxillomandibular fixation for two weeks. However, the advantages are more than the temporary discomfort caused to the patient postoperatively by SSO. This technique needs to be popularised for the excision of deeply seated pathology as there are less postoperative morbidities, like mandibular discontinuity, and there are possibilities of obtaining primary wound healing.

There are case reports by several authors who have described the successful removal of odontomas via unilateral sagittal splitting of the ramus/body of the mandible [3, 8-9].

Thus, in our case, considering the young age of the patient, benign nature of the lesion, the location of the lesion in the posterior mandible, the need for good accessibility, and minimum morbidity by the present approach, we decided to remove the lesion through SSO. By using this technique, we avoided the increased morbidity associated with resection and bone grafting that is undesirable in a young patient. Wound healing was good, and there was no paresthesia in the lip and chin region during immediate and later postoperative periods. The patient was reviewed periodically, and there were no complications associated with the procedure.

In this case, the sagittal splitting of the mandible provided excellent surgical access to a hard tissue mass lying within the mandibular cortices and offered considerable advantages over other more conventional surgical approaches [9].

## Conclusions

SSO can be very useful to access various pathologies in the body, angle, and ramus of the mandible and to navigate lesions which are near the IAN, thus preserving the nerve and also helping to avoid postoperative morbidity when compared to other conventional surgical approaches. It can be used to remove large cysts, benign non-infiltrative tumours of the mandible, odontogenic myxoma, large odontoma and deeply impacted lower third molars.
